# Predicting the future of neuroimaging predictive models in mental health

**DOI:** 10.1038/s41380-022-01635-2

**Published:** 2022-06-13

**Authors:** Link Tejavibulya, Max Rolison, Siyuan Gao, Qinghao Liang, Hannah Peterson, Javid Dadashkarimi, Michael C. Farruggia, C. Alice Hahn, Stephanie Noble, Sarah D. Lichenstein, Angeliki Pollatou, Alexander J. Dufford, Dustin Scheinost

**Affiliations:** 1 Interdepartmental Neuroscience Program, Yale School of Medicine, New Haven, CT USA; 2 Child Study Center, Yale School of Medicine, New Haven, CT USA; 3 Department of Biomedical Engineering, Yale School of Engineering and Applied Science, New Haven, CT USA; 4 Department of Radiology and Biomedical Imaging, Yale School of Medicine, New Haven, CT USA; 5 Department of Computer Science, Yale School of Engineering and Applied Science, New Haven, CT USA; 6 Department of Psychiatry, Yale School of Medicine, New Haven, CT USA; 7 Department of Psychiatry, Columbia University Irving Medical Center, New York, NY USA; 8 Wu Tsai Institute, Yale University, New Haven, CT USA

**Keywords:** Neuroscience, Predictive markers, Psychiatric disorders

## Abstract

Predictive modeling using neuroimaging data has the potential to improve our understanding of the neurobiology underlying psychiatric disorders and putatively information interventions. Accordingly, there is a plethora of literature reviewing published studies, the mathematics underlying machine learning, and the best practices for using these approaches. As our knowledge of mental health and machine learning continue to evolve, we instead aim to look forward and “predict” topics that we believe will be important in current and future studies. Some of the most discussed topics in machine learning, such as bias and fairness, the handling of dirty data, and interpretable models, may be less familiar to the broader community using neuroimaging-based predictive modeling in psychiatry. In a similar vein, transdiagnostic research and targeting brain-based features for psychiatric intervention are modern topics in psychiatry that predictive models are well-suited to tackle. In this work, we target an audience who is a researcher familiar with the fundamental procedures of machine learning and who wishes to increase their knowledge of ongoing topics in the field. We aim to accelerate the utility and applications of neuroimaging-based predictive models for psychiatric research by highlighting and considering these topics. Furthermore, though not a focus, these ideas generalize to neuroimaging-based predictive modeling in other clinical neurosciences and predictive modeling with different data types (e.g., digital health data).

## Introduction

Neuroimaging studies of psychiatry have gained invaluable insight into neural mechanisms underlying psychiatric disorders. These studies range from activation studies to case-control designs to identifying individual differences in behavior. However, bridging the gap between the knowledge that we have gained from these studies to identifying biomarkers for further applications in therapeutic interventions remains a challenge [1, 2]. Predictive modeling with neuroimaging data [3, 4] has the potential to characterize psychiatric disorders and inform clinical decisions. There is a growing body of literature demonstrating the promise of this application of predictive modeling [2, 5–7], explaining the fundamental mathematics underlying machine learning [8, 9], and establishing best practices for using these approaches with neuroimaging data [10–13]. Even more so, there have been many works reviewing previous mental health studies using machine learning and neuroimaging data [6, 10–15].

Instead, as our knowledge of mental health and machine learning continues to evolve, we aim to look forward—or predict—and present a curated set of topics that we believe will be important in current and future studies. We begin by discussing two timely issues from machine learning that may be unfamiliar to the broader psychiatric neuroimaging community: “bias and fairness” and “dirty data.” Next, we highlight that the level of interpretation is crucial for models to achieve their full impact and depends on the goal of the investigation. Finally, we demonstrate a role for predictive models in two popular areas of mental health research: transdiagnostic research and targeting imaging-based brain markers. The target audience of this work is a researcher familiar with the fundamental procedures of machine learning (such as those present in ref. [16], rather than a deep understanding of the underlying algorithms and mathematics) who wishes to increase their knowledge of ongoing topics and issues in the field. Nevertheless, it can also serve as an introduction for beginners to topics not generally covered in depth in previous reviews, as well as a point of discussion for the more experienced. We aim to maximize the utility and applications of neuroimaging-based predictive models for mental health research by highlighting these topics. While examples are rooted in neuroimaging data, these topics and considerations apply to other data forms (e.g., digital health data).

## Section 1: Topics around data representation

We begin by discussing issues in obtaining data and the issues that arise with how subject populations are represented in our samples. Using training data that are not representative of the real-world population of interest is a putative source of bias, which can cause brain-behavior models to be inaccurate in certain demographic groups. Appreciating the potential for bias, uncovering sources of bias, and minimizing the impact of bias are critical. Additionally, we discuss the need to use rich, real-world data with its associated complexities in these models to understand psychiatric disorders, with consideration of consequent issues.

### Bias and fairness

In machine learning, bias is defined as “results that are systematically prejudiced toward an individual or a group based on their inherent and acquired characteristics” [17], whereas fairness is defined as “the absence of bias.” Data used to train the model is often the leading source of this bias. This principle has been recently highlighted by the PULSE AI [18] photo recreation model; the model turned a low-resolution image of former President Barack Obama into an image of a white male. These results are putatively because the underlying algorithm is trained on a limited demographic, leading to accurate predictions only for certain racial groups [19, 20]. In addition, it has been increasingly recognized that a large proportion of academic studies–including studies relevant to psychiatric research–are based on a western, educated, industrialized, rich, and democratic population, as these participants are most conveniently available [21].

Similarly, studies of particular subpopulations, such as individuals with psychiatric disorders, are also limited by subjects’ abilities to be scanned; these limitations may include: subjects’ abilities to remain still during a scan, their ability to consent, or their ability to attend during the scan. Consequently, studying this limited population biases results by under-representing the variance in behavior across the broader population of interest. Additionally, our inability to study these populations also contributes to training a model that is not representative of real-world circumstances and the behavioral variabilities associated with our phenotype of interest. For these reasons, some argue that bias in machine learning is solely rooted in biased data and that algorithms are not biased [22]. However, as bias can stem from nearly limitless sources [23], creating bias-free data may not be possible [24]. Consequently, the simplistic view of “only the data is biased” can undercut biases that stem from machine learning algorithms themselves. Overall, generating and applying predictive models without acknowledging bias can lead to inappropriate overgeneralizations where certain populations are overrepresented, leading to others being underrepresented.

**Fig. 1:**
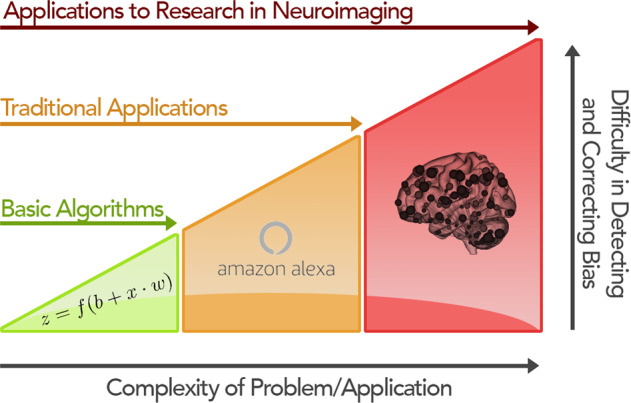
The increasing difficulty of understanding biases as application complexity increases. In theoretical work, such as algorithmic proofs, bias is low, putatively, as these works often do not focus on real-world data. However, biases quickly emerge in well-established applications in machine learning, like language and image processing. These biases may be missed during the initial product development but can quickly become apparent upon widespread use. Finally, for emerging applications of machine learning, such as in psychiatry, potential biases are often hard to observe, understand, and prevent, in part because (1) our knowledge of mental health disorders is still limited in comparison to traditional applications like image processing and (2) the data may not be comprehensive enough to model the complexities of mental health fully.

These issues are exacerbated when predicting psychiatric information from neuroimaging data (Fig. 1). Research on the complexities in neural mechanisms underlying psychopathology is still ongoing, and much of this remains unknown to us [25]. Available data are small compared to traditional machine learning applications and likely, overrepresent specific populations over others. Additionally, while ever-improving, our knowledge of brain-behavior associations remains basic, such that sources of biases may not be readily apparent. Core symptoms and underlying brain circuits that overlap across different mental health disorders [26], evolving knowledge and grouping of symptoms to characterize disorders, and the complexity of real-world data are potential sources of bias (see “Dirty data”). Further, there may be important cross-cultural differences in symptom presentation, as well as differing perspectives on mental health more broadly. Thus, models trained on one demographic group may not generalize to others [3, 4, 19, 21]. A failure to generalize across different populations is not inherently disadvantageous. For example, sex and age differences have been reported in functional connectivity and may warrant different models to predict the same phenotypic measure, reflecting group differences in underlying neural circuitry. Such differences between models do not invalidate any model. Though, it necessitates caution in interpretation and motivates new research [16].

In practice, training algorithms on diverse sets of data, both in size and demographic distribution, offer a practical way to reduce bias. Large open datasets that aim to represent the broader population—i.e., the Adolescent Brain Cognitive Development Study [27] and the UK Biobank [28]—or datasets from multiple countries have the ability to provide a more broadly applicable representation of a disorder. Testing in larger, representative samples is more likely to pick up on generalizable rather than idiosyncratic features, leading to decreased inflation of prediction accuracy [29, 30]. This decrease in accuracy may introduce publication bias as researchers could forgo this step to improve effect sizes. While there is no current solution for biases, it is important to recognize that innate biases exist and that caution should be taken in study design and result interpretation [31].

### Dirty data

Dirty data, as we refer to it here, is characterized by complexities introduced by missing, inaccurate, incomplete, or inconsistent data [32]. Dirty data is present in many, if not all, neuroimaging studies related to mental health research. (Table 1) However, specifically for predictive models and their subsequent clinical utility, models must work on real-world data, which is complex and noisy. While dirty data is often conceptualized as a shortcoming in research, the complexities and richness of data in psychiatry are intrinsic and must be carefully considered rather than immediately eliminated. Only by fully appreciating these factors can predictive models fulfill their promise of clinical application.

**Table 1:** Sources of dirty data.

Category	Problem	Examples
Phenotypic measures	Measures are subjective	• Poor inter-rater reliability and high variability in gold-standard diagnostic tools and behavioral measures [33–35, 81]
	Measures are nonspecific	• High false-positive rate on ADOS in adults with schizophrenia [36]
	Measures focus on the tails of behavior	• Healthy controls will be zero inflated on questionnaire data [37, 38]
Participants	Comorbidity	• Symptoms of psychiatric disorders often overlap across diagnoses, while the majority of predictive models in psychiatry rely on more binary classification approaches
	Medication	• Psychiatric medications have the ability to alter BOLD signal patterns. This becomes difficult to study the psychiatric phenomena of interest as signals are confounded
	Episodic symptoms	• Symptoms change as a function of disease state. Data from scans based on one day may be vastly different in brain states relative to scans based on another day
Data collection	Multi-site	• Inter-scanner differences can induce significant variability [82, 83], and the complexity of the data analysis workflows could affect reproducibility [84]
	Missing data	• Subjects not completing questionnaires • Inability to complete behavioral testing or scan sessions in clinical populations [85, 86]

Subjectivity in phenotypic measures is a source of noise, where this noise introduces the inherent variability of these measures to capture the desired behavior accurately. Thus, subjective variability exists even with expert-trained testing administrators [33–35]. Additionally, while measures can be good at distinguishing pathology from healthy individuals, they are often nonspecific between different psychiatric disorders. For example, there exists a high false-positive rate of an autism diagnosis in adults with schizophrenia based on the Autism Diagnostic Observation Schedule [36]. Relatedly, these phenotypic measures present a further challenge when predicting in a transdiagnostic manner across health and disease (see “Transdiagnostic prediction”). Many current measures used in mental health are often skewed to focus on specific tails of a distribution (i.e., elevated symptoms) [37, 38], leaving little spread in the “healthy” range. But for predictive modeling across a broad population, phenotypic measures should assess the entire distribution, and newer end-points such as Extended Strengths and Weaknesses Assessment of Normal Behavior [39] and digital phenotyping [40] might be needed. As predictive models are only as useful as the quality of their input data, these measures may not reflect the desired end-point for a predictive model. However, these behavioral measures largely serve as the field standard for phenotypic characterization despite these inherent issues. In fact, improving the quantification of complex behavior, as opposed to improving neuroimaging data and prediction algorithms, may lead to the most significant gains in algorithm performance. Accordingly, other approaches for phenotypic characterization are gaining traction but are still nascent in prediction studies. Thus, finding the best end-point for prediction remains a challenge [5].

Given considerable underlying biological heterogeneity in mental health, patients themselves add substantial complexity to the data, with researchers often turning toward small, highly homogenous cohorts to minimize heterogeneity. Aligned with this, the majority of neuroimaging predictive models in psychiatry to date have relied on binary classification approaches (i.e., does this participant have a disorder or not?) [41]. However, comorbidity among psychiatric disorders is common, with estimates greater than 50% [42]. Simple classification approaches face limits as our diagnostic labels are poor due to this heterogeneity. Further, any mental health disorders are episodic, and classification labels/results may change depending on disease state. However, neuroimaging predictive models that adopt transdiagnostic and dimensional approaches have the potential to appropriately address comorbidity [43] by examining the generalizability of common and unique associations with symptoms (see “Transdiagnostic prediction”). Moreover, many patients take daily medications, which may alter the BOLD signal measured by fMRI [44]. Therefore, predictive models may be confounded by “learning” medication artifacts instead of changes in neurobiology underlying the disorder, limiting their potential for both neurobiological interpretation and real-world promise (i.e., classifying an individual as a patient because they take medication is obvious). Nevertheless, the strength of predictive modeling is the ability to train on one population (e.g., participants taking medication or with comorbidities) and test in another (e.g., medication-naive participants or patients without comorbidities). Models that generalize across both patient populations are likely free of said confounds.

Finally, issues around data collection can dirty the data. There is increasing interest in large-scale consortium studies to increase sample sizes, patient diversity, and generalization. Despite efforts to harmonize data, inconsistency exists, and combining datasets can be challenging [45]. Similar to above, holding out sites or even whole studies as testing data and showing that a model generalizes across sites/studies provides strong control for possible inconsistencies. Missing data are another real-world data collection problem. Increasing the amount of data (either neuroimaging or phenotypic) per participant can improve the accuracy of predictive models [7, 46]. However, as the amount of data increases, the chances of missing a portion of data also increases. A common approach to handling missing data is to exclude participants with missing data. However, since data is challenging to collect and is more commonly lost in clinical populations, this approach is suboptimal and can introduce biases to models (see “Bias and fairness” for how excluding specific participants can bias models). Data imputation, or the process of replacing missing data with substituted values, has great promise to improve predictive performance [47–49] by retaining participants with missing data in analyses and, thus, increasing sample sizes. Data imputation is sensitive to the structure of the data, missing data patterns and mechanisms, the machine learning algorithms, and the metrics for model performance [50] and cannot be performed blindly. For example, individuals with more severe symptoms are more likely to fail quality controls. As many data imputation algorithms do not work for data “not missing at random”—or, when the probability of missing data varies for unknown reasons, imputing these data may introduce an additional source of dirtiness and bias. Altogether, accounting for the complexities created by data collection issues can increase sample sizes, which, in turn, can lead to better-performing models.

As data are inherently dirty, over-controlled studies with small sample sizes will ultimately overestimate effect sizes that one can reasonably expect in real-world situations [51]. Leaning into this dirtiness will ultimately better capture the nuanced nature of the brain-phenotype association. Given the ultimate goal of predicting individual values in data not previously seen, predictive modeling offers a natural approach to investigate if a model (i.e., brain-phenotype association) generalizes over these complexities.

## Section 2: Interpretation or prediction

**Fig. 2:**
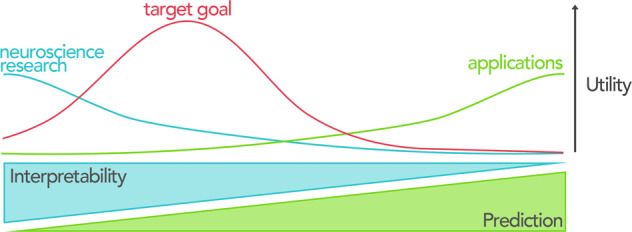
Benefits and trade-offs in using different models. While exceptions may exist, the interpretability of models usually occurs at the price of prediction performance and vice versa. In neuroscience research, including understanding the neural circuits underlying psychiatric disorders, interpretability (defined as the ability to understand the cognitive and neurobiological underpinnings of a model’s features) offers the greatest utility. In contrast, prediction performance is a priority in many real-world products and applications. We argue that the target goal for neuroimaging-based predictive models on the interpretability/prediction performance trade-off is on the interpretability side. At the moment, interpretable predictive models are expected to better advance neuroimaging research in psychiatry and complement traditional approaches than models that sacrifice interpretation for prediction performance.

Models are complex in nature and imperfect in performance and information extraction. As such, there is often a trade-off between a model’s interpretability and prediction performance. Models with high prediction performance tend to operate like a black box where the inputs and outputs are interpretable, but how the model itself works remains unclear. On the other hand, placing emphasis on the interpretability of features usually comes at the cost of the model’s prediction performance, even though features are more (Fig. 2). The desired point on this spectrum depends on one’s goals. For real-world applications (i.e., commercial products), high prediction performance is generally needed. However, given that even the best performing neuroimaging-based models show modest prediction performance, an emphasis on interpretability may be warranted.

In machine learning, interpretability is defined as “the degree to which a human can understand the cause of a decision” [52]. For neuroimaging, this definition can be further refined to “can we understand the regions, connections, networks, or cognitive circuits that lead to predicted values?”. In other words, for neuroscientific interpretation, simply knowing what combinations of brain features predict is not enough. Understanding how these features relate to underlying cognition and neurobiology is likely more useful; thus, sacrificing prediction performance for interpretability (such as using simpler linear models) may be more advantageous. Because we do not fully understand the neural circuitry disruptions and abnormalities in psychiatric disorders, a model aimed toward interpretability can still inform the neural circuits and their disruptions in a particular disorder even with modest prediction performance. However, uninterpretable black box models that do not meet real-world utility have few redeeming qualities in that they can be used neither as an application nor for neurobiological insights.

The high dimensional and noisy nature of neuroimaging data can make neurobiological interpretations difficult, even when a model is interpretable from a machine learning point of view. Features (e.g., functional connections, morphometry, or activation patterns) across the brain are highly correlated, giving rise to issues for feature selection algorithms. For example, using approaches designed to account for high correlations among features (e.g., $\ell ^2$ penalty, principal component regression) can lead to dense models with many features. Likewise, enforcing sparsity by discarding a portion of highly correlated features can lead to model instability [53]. Relatedly, features at the smallest scale (voxels, vertices, or connections) are noisy with low test-retest reliability and contain shared information with neighboring voxels, both of which cause instability. As such, selected features may change with using different iterations of the training data, hurting interpretability [54]. A solution to increase interpretability for both dense and unstable models is to examine a model at multiple levels of summarizations [5]. For example, models based on functional connectivity could visualize the individual connections that comprise a model (i.e., edge-level) while also summing the model weights over each brain region (i.e., node-level) and canonical functional networks (i.e., network-level). Averaging features over regions or networks reduces the amount of noise and redundant sources of information, leading to more stable interpretations of the underlying anatomy and supporting modern systems-level interpretations of the brain.

Dirty data and data quality can also influence where a model falls in the trade-off between interpretability and prediction performance. Putative confounds, such as head motion or aliasing of respiratory and cardiac signals into fMRI data, are often correlated with symptom severity [55, 56], such that those with the highest symptoms have worse data quality. It can be debated whether these factors are “signal” or “noise” as it depends on the goals. For a model prioritizing performance, leveraging these differences may improve prediction performance at the expense of neurobiological interpretation. In contrast, removing these confounds will likely improve neurobiological interpretation at the expense of prediction performance. Nevertheless, when controlling for these or other covariates, training and testing data must be kept independent (see, for example, Rule #2 from [16]). For example, simply regressing a covariate from all data before separation into training and testing data will lead to information leakage between training and testing data and can inflate prediction performance.

Perhaps, given the discussion above, the most interpretable analyses are statistical inference methods that do not focus on out-of-sample prediction. If so, why use predictive models at all? Mental health disorders are complex in nature, and beyond the neural substrates underlying mental health disorders, there remains a myriad of contributing factors we have yet to understand. The answer may be as simple as out of sample testing (i.e., prediction) being better at handling these dirty, real-world data than many traditional approaches (see “Dirty data” and “Transdiagnostic prediction”). Additionally, a focus on only explaining a particular theory or mechanism gives little knowledge on how that may predict future outcomes, a putative weakness of understanding of behavior [30]. Overall, interpretable predictive models can complement explanatory models by filling in gaps left unexplained by explanatory models.

## Section 3: Topics in psychiatric applications

Finally, we discuss a role for predictive models in two popular topics in psychiatry: transdiagnostic research and targeting imaging-based brain markers. While significant discussion surrounding these topics in psychiatry exists [11, 14, 57], neuroimaging-based predictive models offer unique insights into these topics. Predictive modeling naturally lends itself to analyzing data in a transdiagnostic manner. These methods are routinely used to cluster individuals based on biological factors (e.g., neuroimaging data) rather than symptoms. But, as detailed below, there are other ways to use these approaches for transdiagnostic research. Finally, we review the emerging literature to emphasize how even complex neuroimaging-based predictive models can be targets for interventions.

### Transdiagnostic prediction

**Fig. 3:**
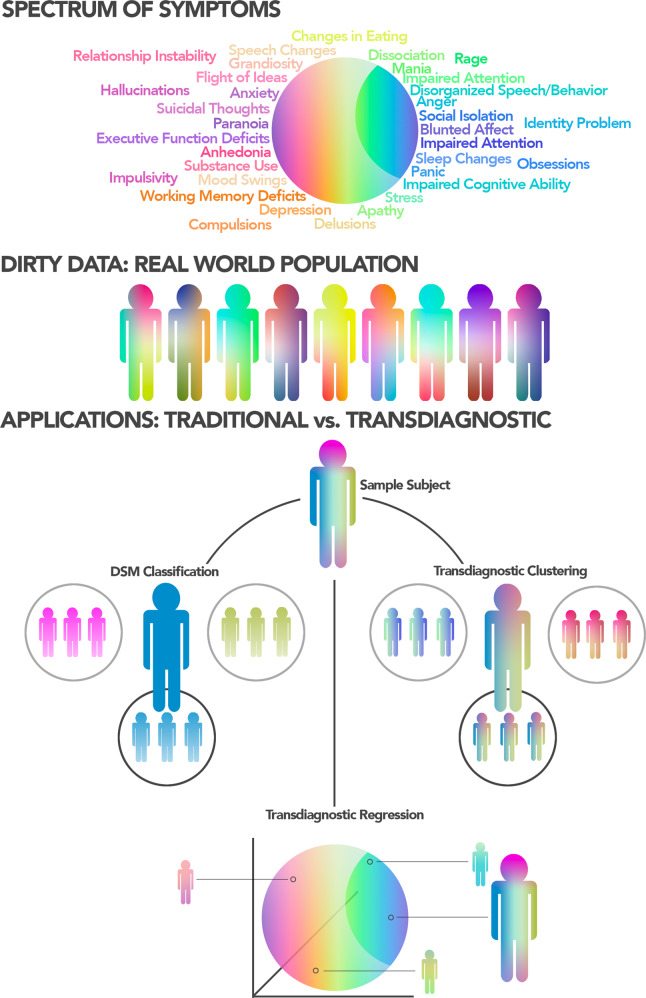
Transdiagnostic prediction. Current theories postulate that symptoms lie on a continuum, where distinct symptoms group together in overlapping clusters. As a result, and as discussed in “Dirty data”, real-world patients often exhibit many different patterns of symptoms and comorbidities rather than a single distinct pattern. Such viewpoints make classification into textbook diagnoses difficult as these diagnoses are based on meeting exemplar symptom patterns. Predictive models offer a solution to transdiagnostic problems, either by placing an individual into a cluster of patients that most mimic their spectrum of symptoms (i.e., transdiagnostic clustering) or by identifying brain networks that predict symptoms and generalize across a spectrum of traditional clinical categories and “healthy” individuals (i.e., transdiagnostic regression).

The introduction of the NIMH Research Domain Criteria [25] has helped popularize the idea of transdiagnostic research in psychiatry. Transdiagnostic research aims to eschew traditional diagnostic categories, instead representing individuals along behavioral and biological spectrums encompassing both patients and individuals with subclinical symptoms. Such approaches can cause problems for traditional inferential methods (e.g., ANOVAs), where statistical power is formed by having homogeneous groups of individuals that are maximally separated from each other. Nevertheless, to best capture symptoms along a spectrum and replicate real-world circumstances (see “Dirty data”), the separation between groups should not be maximized but rather minimized. Predictive models handle this type of data well [2–5, 7] and, thus, allow us to take a transdiagnostic approach toward psychopathology that appreciates the heterogeneity of symptoms within and across patients and “healthy” individuals (Fig. 3). As such, transdiagnostic models putatively find more generalizable brain features underlying behavior–rather than idiosyncratic ones fit toward a specific disorder.

Currently, the most popular form of transdiagnostic predictive modeling aims to account for the heterogeneity in mental health by clustering individuals into smaller subgroups based on biological measures (e.g., neuroimaging data) rather than predetermined collections of symptoms (for example, from the DSM-5 [58]). These promising approaches aim to overcome the limitation of traditional classification approaches where labels are poor (see “Dirty data”). Increasing evidence suggests that these data-derived subgroups, which cut across diagnostic categories, offer better labels to train on, leading to improved prediction of treatment outcomes [59]. Accordingly, neuroimaging data have been the primary data type for this transdiagnostic investigation [59–65]. Additionally, semi-supervised learning, where labeled and unlabeled data are used jointly, may represent a promising approach to account for heterogeneity in mental health [66, 67]. Nevertheless, its goal of creating biologically driven diagnoses can also be viewed as a weakness in that individuals are still stringently categorized into groups rather than treating everyone on a spectrum. Fuzzy clusters with overlapping boundaries between groups can ameliorate this weakness, possibly even allowing an individual’s probability of membership to shift as a function of disease state (e.g., depression, mania). Moreover, when treating patients, clustering that individual into a definitive group may offer the most desired outcome as it may provide the clearest blueprint for treatment. However, this is only optimal when there are clear differences in treatment efficacy across these derived groups.

An alternative approach is to predict continuous phenotypic measures across a sample from diverse mental health backgrounds with the aim of identifying a “transdiagnostic” network generalizable across traditional clinical categories [68]. Examples of this approach include training a model on patients with one disorder and testing it on patients with a different disorder; training a model of behavior in healthy individuals and testing to see if it generalizes to pathology; or training a model of symptom severity across multiple diagnostic groups. This approach has been unfavorable in the past due to uncertainty about the specificity of an effect and a desire for tightly controlled diagnoses. For example, when mixing participants with psychiatric disorders and controls for comparisons, the degree to which outcomes are driven by patients relative to controls is unclear. Alternatively, given a mix of patients with different mental health backgrounds, it can be unclear whether models truly measure the desired phenotype exclusively or are driven by unmeasured confounds. Nevertheless, if symptoms a) range from average to subclinical to clinical, b) can dynamically increase and decrease as disease state changes, and c) putatively rely on the same brain circuits, this type of approach is needed. Given the strengths of predictive modeling to train on one population (e.g., healthy individuals) and test in another (e.g., patients), these questions can be systematically addressed in a step-wise manner by repeatedly testing and training in different combinations of diagnostic categories [69]. Such approaches also eliminate the need to place an individual into a specific group--whether data-driven or classically derived. The model itself will capture relevant brain features about the phenotype being predicted. Nevertheless, these studies are only emerging, and their ultimate utility is still to be determined [69–71].

### Targeting model features

Given the distributed, whole-brain nature of neuroimaging-based predictive models and the resulting challenges to interpretation (see “Interpretation or prediction”), questions remain regarding how these results can be implemented in clinical practice and even targeted by interventions. The most direct cases of using predictive modeling in clinical practice would be using a case-control classification model for diagnostic purposes or a transdiagnostic classification model to sub-type an individual into the intervention with the highest likelihood of success [62]. However, most classification models do not have sufficient accuracy in, and generalization to, real-world data. As such, showing that brain features underlying a model can be targeted and modulated by a potential therapeutic approach provides a translational avenue for predictive models. In other words, the most promising models currently available might not be simply the ones that perform the best, but the ones that can be targeted by current or novel interventions. A range of works is emerging in this area.

Given that pharmacology is a front-line treatment for most psychiatric disorders, showing that the effect of a particular medication is specific to the underlying brain features of a predictive model provides both an external validation of the model and a potential marker to compare competing medications individually. For example, the medication that best modulates brain features associated with the improvement of symptoms potentially represents the best medication to treat those symptoms. As predictive models operate at the individual level, this process could help find the best medication for the patient at hand, given a range of potential medications. Indeed, initial data suggests that whole-brain predictive modeling approaches can be effectively modulated with medication [72]. Using a predictive model of sustained attention—an executive function that is reduced in individuals with attention deficit/hyperactivity disorder (ADHD)—it was shown that a single dose of methylphenidate, a stimulant medication commonly used to treat ADHD, specifically changed functional connectivity within that model. While preliminary, these results open a novel line of research centering around predictive modeling and pharmacological MRI aimed at ameliorating psychiatric symptoms.

In addition to pharmacological treatment, predictive models can directly inform newer experimental therapies. For example, neurofeedback (via EEG, fNIRS, or fMRI) is a neurotherapeutic approach where an individual learns to modify their brain function to optimize symptoms, cognition, or behavior [73]. A challenge of neurofeedback is finding the correct set of brain features for an individual to learn to control. Putatively, these features should strongly relate to, or perhaps better predict, the behavior that is to be improved, such that exerting control over these features will lead to a change in behavior. It is increasingly recognized that many symptoms or behaviors cannot be localized to a single region [74] but instead rely on the orchestrated activity of a distributed array of regions [75, 76]. As such, pilot studies are starting to use complex, whole-brain models trained on independent samples of participants as targets for neurofeedback [77, 78]. Nevertheless, this promising approach has only been applied in a few studies and its efficacy remains unknown.

Finally, like neurofeedback, neuroimaging-based predictive models have been used to inform which site to target using transcranial magnetic stimulation (TMS). Connectivity models based on normative databases have been used to individualize targets for TMS, producing dramatic increases in treatment response [79]. Moreover, researchers applied predictive modeling with independent samples of individuals who received TMS for major depression to identify distinct TMS targets associated with improvements in dysphoric vs. anxiosomatic depressive symptoms [80]. These results highlight the potential of using whole-brain predictive modeling approaches to individualize neuromodulatory targets based on symptom presentation and/or individual neuroanatomy, as well as to identify novel TMS targets for clinical applications.

Collectively, there is ample evidence to illustrate that there are a variety of promising approaches to target model features, all of which have great potential to improve existing treatments and facilitate the development of novel treatment approaches.

## Conclusion

The use of neuroimaging-based predictive models is becoming increasingly common in psychiatric research. While these are powerful tools to analyze neuroimaging data, both the fields of machine learning and psychiatry continue to evolve, adding further complexities to current best practices [3, 4]. In this work, we detail topics that we believe to be important in current and future studies, but that also may be less familiar to the broader community using neuroimaging-based predictive modeling in mental health research. Furthermore, we anticipate that many of these ideas will generalize to neuroimaging-based predictive modeling in the context of neurological disorders and to predictive modeling using other data types in psychiatry. Careful consideration of these emerging topics in machine learning and psychiatry will help researchers best apply neuroimaging-based predictive models to push forward our understanding of mental health.
